# Cerebral Computed Tomographic Findings in Schizophrenia: Relationship to Second-Generation Antipsychotics and Hyperprolactinemia

**DOI:** 10.3390/healthcare12131343

**Published:** 2024-07-05

**Authors:** Paula Simina Petric, Petru Ifteni, Andreea Violeta Popa, Andreea Teodorescu

**Affiliations:** 1 Facultatea de Medicină, Universitatea Transilvania din Brașov, Bulevardul Eroilor 29, 500036 Brașov, Romania; paula_petric@yahoo.com (P.S.P.); andreea_popa32@yahoo.ro (A.V.P.); andre_martie@yahoo.com (A.T.); 2 Spitalul Clinic de Psihiatrie și Neurologie Brașov, Str. Prundului No. 7-9, 500123 Brașov, Romania

**Keywords:** prolactinomas, neuroimaging, cerebral CT (computed tomography), antipsychotic side effects, schizophrenia, hyperprolactinemia

## Abstract

Antipsychotic medications are essential for managing severe mental illnesses like schizophrenia, which impacts about 1% of the global population. Despite efficacy, in some cases, they can induce hyperprolactinemia, affecting roughly half of the patients. The prevalence of this condition varies with the specific medication used. Although prolactinomas are rare among schizophrenia patients, treating them with dopamine agonists poses conflicts with antipsychotic medication, necessitating careful monitoring and adjustments. The aim of this study was to explore the presence of brain tumors, prolactinomas, and other structural brain changes in schizophrenia patients treated with second-generation antipsychotics using cerebral computed tomography (CT) scans. We conducted a cross-sectional study involving 152 hospitalized patients diagnosed between 1 January 2020 and 31 March 2024. Evaluations included cerebral CT scans, prolactin level assessments, and the monitoring of side effects. Patients, with an average age of 42.79 years and an illness duration of 17.89 years, predominantly received olanzapine (46.05%) and risperidone (36.84%). Side effects, reported by 61.78% of patients, included tremors, dizziness, and weight gain. Abnormal prolactin levels were observed in 53.95% of patients, more prevalent in females on risperidone and in both genders on olanzapine. No prolactinomas were detected on CT scans. Managing hyperprolactinemia in schizophrenia patients undergoing antipsychotic therapy is essential to prevent long-term complications and to ensure treatment compliance.

## 1. Introduction

Schizophrenia is a complex mental disorder characterized by symptoms that disrupt daily life, such as hallucinations, delusions, and disorganized thinking [1]. These challenges arise from structural and functional changes in the brain, including reduced gray matter volume and disruptions in neural circuits, particularly in areas like the prefrontal cortex [2,3]. Given the severity of these symptoms and their impact on individuals’ well-being, effective treatment is very important [4].

Antipsychotic medication is the primary approach to managing schizophrenia, with both first-generation (typical) and second-generation (atypical) options available [4]. Some studies indicate that antipsychotic medications, particularly first-generation ones, may be associated with a reduction in gray matter volume, raising concerns about potential neurotoxic effects [5]. However, second-generation antipsychotics might have a less pronounced effect on gray matter reduction or even mitigate it in some cases [6]. Additionally, there is an ongoing debate about whether antipsychotic medications have a neuroprotective effect by reducing psychosis-related harm to the brain or whether they contribute to brain volume reductions observed in long-term use [7].

Prolactin is produced by pituitary lactotroph cells, regulated by dopamine released from neurons originating in the hypothalamic arcuate and ventromedial nuclei. Unlike other pituitary cells, lactotrophs exhibit high basal secretory activity, with dopamine exerting a continuous inhibitory influence on prolactin secretion by binding to D2 receptors on the cell surface. A reduction in dopamine control leads to a swift rise in prolactin secretion [8]. Antipsychotic medications induce hyperprolactinemia by blocking the D2 receptor. Many first-generation antipsychotics act as strong antagonists of the D2 receptor, although their affinity and potency towards this receptor can vary [9]. While some may strongly bind to the receptor, inducing prolonged D2 blockade and potentially impacting prolactin levels over time, others may exhibit different degrees of interaction with the receptor. Haloperidol, flupentixol, and zuclopenthixol induce hyperprolactinemia by blocking dopamine D2 receptors in the pituitary gland. This blockade removes the inhibitory effect of dopamine, leading to increased prolactin secretion and elevated prolactin levels in the blood, with haloperidol being the most commonly associated with this side effect due to its potent dopamine D2 receptor antagonism [10,11]

In contrast, most second-generation antipsychotics detach from the D2 receptor more rapidly. For example, in both quetiapine and clozapine, receptor occupancy decreases from 60–70% at 2 h post-dose to less than 30% at 24 h, at least in the brain [12]. Risperidone and paliperidone induce hyperprolactinemia by blocking dopamine D2 receptors in the pituitary gland, disrupting dopamine’s inhibitory effect on prolactin secretion [13,14]. Aripiprazole, however, as a partial agonist at dopamine D2 receptors, maintains dopamine activity, reducing the risk of hyperprolactinemia. Its unique pharmacological profile, including partial agonism at serotonin 5-HT1A receptors and antagonism at serotonin 5-HT2A receptors, contributes to this reduced tendency [15,16].

Antipsychotic medications often lead to hyperprolactinemia in patients, with around 50% of those prescribed experiencing the condition, compared to just 0.4% of the general population [17]. A review of 14 cross-sectional studies involving 2235 individuals on antipsychotic medication revealed that hyperprolactinemia prevalence varied widely, ranging from 42% to 93% in women and 18% to 72% in men [18]. Women of reproductive age, especially those who have given birth, seem to face a higher risk of hyperprolactinemia compared to post-menopausal women. However, in one study conducted for over one year, examining the changes in prolactin levels among first-episode schizophrenia patients treated with one of four atypical antipsychotics (olanzapine, quetiapine, amisulpride, ziprasidone), it was observed that the administration of these medications did not lead to a significant increase in prolactin levels in either men or women [19]. Although data are limited, adolescents also appear susceptible to developing hyperprolactinemia [20,21].

Hyperprolactinemia can lead to a variety of physical and behavioral effects. Physically, it often causes galactorrhea, irregular menstrual cycles in women, and erectile dysfunction in men. It can also result in decreased libido, infertility, and osteoporosis due to the disruption of other hormone balances. Behaviorally, individuals may experience mood disturbances, anxiety, and depression [22].

The incidence of hyperprolactinemia varies significantly among different antipsychotic medications. For first-generation antipsychotics, reported prevalence ranges from 33% to 87%, depending on the dosage. Among second-generation antipsychotics, amisulpride demonstrates the highest potential for increasing prolactin levels. Even at relatively low doses (50 mg/day), nearly all patients experience hyperprolactinemia. Risperidone (72–100%) and paliperidone also exhibit high rates of hyperprolactinemia. In contrast, other SGAs are associated with much lower rates. Aripiprazole has the lowest rates (3.1–5%), while clozapine, a relatively weak D2 antagonist, leads to hyperprolactinemia in less than 5% of cases. Hyperprolactinemia occurs in 0–29% and 6–40% of individuals taking quetiapine and olanzapine, respectively [23].

**Figure 1:**
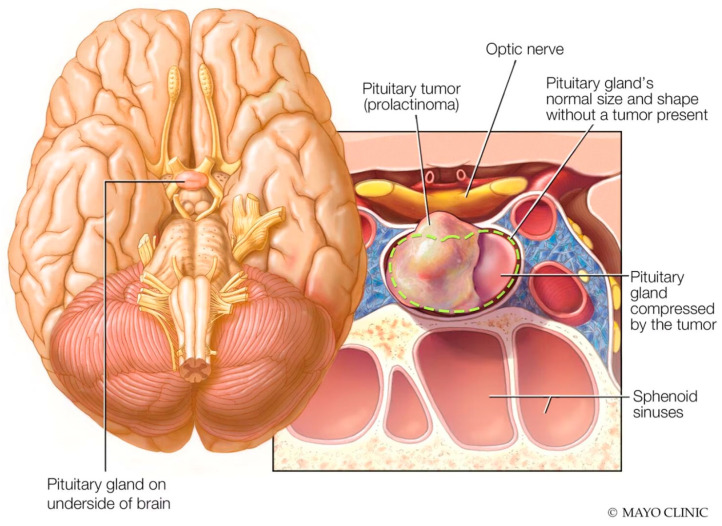
Anatomical location of prolactinoma in the pituitary gland (Image source: https://www.mayoclinic.org/diseases-conditions/prolactinoma/symptoms-causes/syc-20376958, accessed on 17 June 2024).

Prolactinomas, benign tumors originating in the pituitary gland, can develop due to various factors such as hormonal imbalances, genetic predispositions, or specific medications. While relatively uncommon in the general population, with a prevalence of approximately 50 per 100,000 and an annual incidence of 3–5 new cases per 100,000 individuals, their occurrence among patients with schizophrenia is particularly significant due to the potential influence of antipsychotic medications [24]. Since many antipsychotic drugs, especially first-generation ones, can trigger hyperprolactinemia by blocking dopamine receptors, individuals with schizophrenia may face an increased risk of developing prolactinomas [25,26]. These tumors are commonly identified through imaging techniques such as computed tomography (CT) scans. On a CT scan, these tumors typically appear as well-defined masses within the pituitary gland, often leading to the enlargement of the sella turcica (Figure 1) [27,28].

Although prolactinomas are rare in patients with schizophrenia, managing them can be challenging due to concurrent antipsychotic medication use. The primary treatment for prolactinomas involves dopamine agonists such as cabergoline or bromocriptine, which reduce prolactin secretion and tumor size. However, these medications can interact with antipsychotic medications, potentially elevating prolactin levels by blocking dopamine receptors. As a result, schizophrenia patients on antipsychotic medication may experience difficulties with hyperprolactinemia. They may require careful monitoring and potential adjustments in medication regimens to achieve symptom control while minimizing adverse effects [29].

Schizophrenia is associated with a significantly increased risk of premature mortality, with individuals diagnosed with the disorder facing a reduced life expectancy of 15–20 years compared to the general population. The elevated mortality rates in schizophrenia are primarily attributed to a higher prevalence of cardiovascular disease, metabolic disorders, and substance use disorders among patients. Poor lifestyle choices, such as smoking, sedentary behavior, and inadequate healthcare access, further exacerbate the mortality risk in this population [30].

The aim of this study was to explore the presence of brain tumors, prolactinomas, and other structural brain changes in schizophrenia patients treated with second-generation antipsychotics using cerebral computed tomography (CT) scans.

## 2. Materials and Methods

### 2.1. Study Design and Setting

We conducted a cross-sectional study analyzing cerebral CT examinations, prolactin levels, and antipsychotic side effects in patients diagnosed with schizophrenia and treated with atypical antipsychotics from 1 January 2020 to 31 March 2024. The study was conducted at the Clinical Hospital of Psychiatry and Neurology in Brasov, Romania. The hospital is an academic setting with 150 beds for acute patients and 300 beds for chronic patients, serving a heterogeneous population of more than 500,000 inhabitants, including individuals of Romanian, German, and Hungarian ethnicity.

### 2.2. Participants

Inclusion criteria were: patients aged between 18 and 45 years, diagnosis of schizophrenia according to DSM-5 criteria; patients receiving treatment with a single second-generation antipsychotic; patients who underwent a cerebral CT examination during their hospitalization for reasons such as differential diagnosis, headache, loss of consciousness, dizziness, vision disturbances, or balance disturbances; and prolactin level assessment at least 7 days after any change in dosage or type of antipsychotic treatment during their hospitalization.

Exclusion criteria included patients who did not provide written informed consent upon admission, were outside the age range of 18 to 45 years, had diagnoses other than schizophrenia, received treatment with more than one antipsychotic or medications affecting prolactin levels, had a history of alcohol or drug abuse or head trauma, were diagnosed with known cerebral tumors or prolactinomas, or had conditions known to elevate prolactin levels such as pregnancy, breastfeeding, hypothyroidism, or chronic kidney disease.

**Figure 2:**
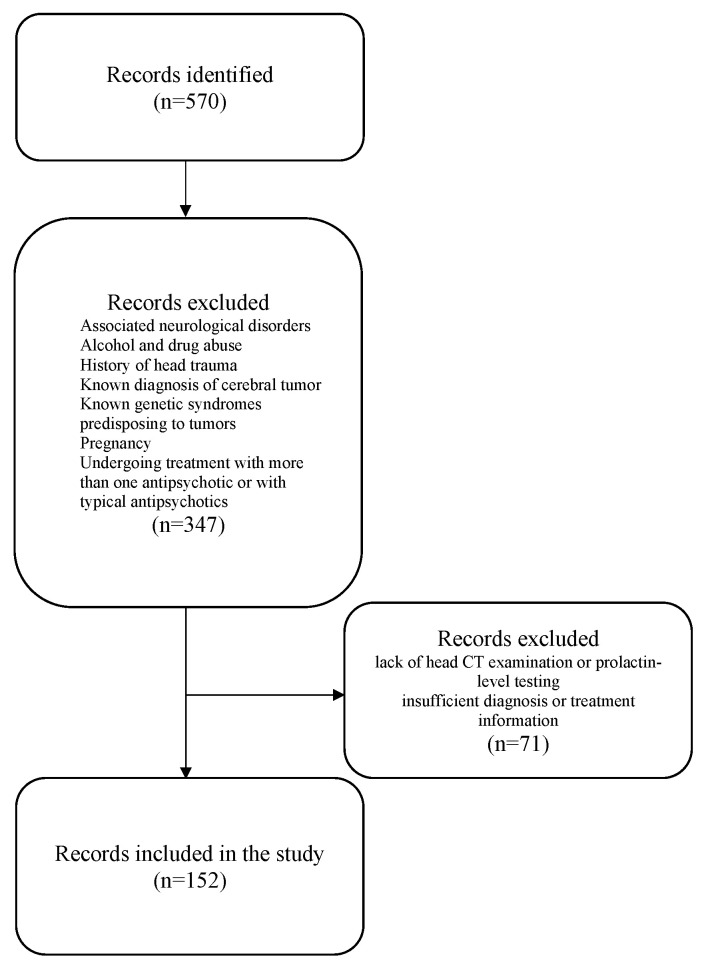
Study flowchart.

During the study period, 570 individuals with schizophrenia were identified as potential participants. Out of these, 347 were excluded because they did not meet the specific inclusion and exclusion criteria. The final analysis included 152 hospitalized schizophrenia patients who met the inclusion criteria (Figure 2).

### 2.3. Data Collected:

The data were collected from both paper and electronic records of patients, including basic demographic information, treatment details such as type, formulation, dosage, side effects, cerebral CT examinations, and prolactin levels.

CT scans were performed in the hospital’s imaging department using the Somatom Spirit apparatus, identified by number 10,165,566 and classified as class IIb. Operating at a minimum frame frequency of 10 frames per second, each examination produced a total of 1100 images, the resolution is 15.5 lp/cm. CT examinations included various measurements, such as the transverse diameter between the tips of the frontal horns on the axial section, the transverse diameter at the lateral ventricle bifurcation at its most cranial point on the axial section, the transverse diameter at the level of the diencephalic third ventricle on the axial section, the anteroposterior diameter at the level of the fourth ventricle on the axial section, and density measurements in both the anterior and posterior portions of the frontal lobe at the cortical and subcortical levels. An experienced imaging specialist conducted the interpretation of the CT scans.

For prolactin level testing, the laboratory defined normal values as 2.80–29.20 ng/mL (59.4–619.04 μUI/mL) for adult women and 2.10–17.70 ng/mL (44.52–375.24 μUI/mL) for adult men. Prolactin levels were measured at least 7 days after any change in the dose or type of antipsychotic medication. All information regarding prolactin levels was recorded in both the electronic and paper records of the patients.

### 2.4. Ethical Considerations

This study was approved by the hospital ethics committee, decision no. 6 on 18 December 2018, and was part of a doctoral research project. The approval included prospective and retrospective imaging studies, cognitive assessments, and the evaluations of the side effects associated with antipsychotic medication. Some of the study objectives were affected by the COVID-19 pandemic.

### 2.5. Statistical Analysis

A statistician conducted the statistical analysis, employing a range of tests to assess the study data thoroughly. We used Student’s *t*-test to determine statistical significance for comparing means and the chi-square test for comparing proportions. All statistical tests adhered to a *p*-value of 0.05. The statistical software SPSS version 20.00 was instrumental in executing these analyses, providing a robust framework for interpreting observed differences and relationships within the dataset, thereby ensuring the reliability of study findings.

## 3. Results

### 3.1. Patient Characteristics

Upon analyzing the demographic characteristics, it was observed that the cohort had an average age of 42.79 (±11.66 SD) years, of which 39.04% were male participants. The average duration of illness was 17.89 years (±11.56 SD) with an average of 25 hospitalization episodes throughout the illness (Table 1).

Table 1 also presents the distribution and dosage characteristics of various antipsychotic medications. Notably, olanzapine was the most frequently prescribed antipsychotic, constituting 46.05% of the cohort, with a mean dosage of 14.5 mg/day (±4.43 SD). Risperidone was prescribed to 36.84% of the patients, with a mean dose of 3.33 mg (±1.61 SD), and under 10% of the patients received treatment with amisulpride, aripiprazole, quetiapine, paliperidone, and clozapine. 

In both male and female patients, the most commonly prescribed antipsychotic treatment was olanzapine, with 43.86% of the male patients and 47.37% of the female patients receiving it. Following olanzapine, risperidone was the second most prescribed treatment in both groups, with 40.35% male and 34.73% female patients being prescribed it (Table 2).

The chlorpromazine equivalent was determined using the reference system outlined in Table 3. This method serves to compare the effectiveness of various antipsychotic medications. Chlorpromazine, among the earliest antipsychotic medications developed, acts as a standard against which the potency of others is measured. Expressing the dosage of different antipsychotic medications in terms of chlorpromazine equivalents offers a clearer understanding of their relative efficacy, helping clinicians in dosage adjustments as necessary. This approach promotes standardized and comparable treatment protocols across different medications, ensuring optimal patient care [31,32]. In our study, amisulpride and quetiapine had the highest mean doses of antipsychotic medications prescribed, while aripiprazole and olanzapine had the lowest mean doses.

### 3.2. Antipsychotic Medication Side Effects

The side effects of the antipsychotic medication were extracted from patient files and categorized using the Uku Side Effect Rating Scale as reference [33]. These adverse effects were broadly divided into four categories: psychic, neurological, autonomic, and other side effects. Psychic side effects included concentration difficulties, asthenia, sedation, memory impairment, depression, inner tension/restlessness, changes in sleep duration and dream activity, and emotional numbness. Neurological side effects included dystonia, rigidity, decreased or increased movement, tremors, akathisia, seizures, paresthesia, and headaches. Autonomic side effects involved disturbances in accommodation, salivation changes (either increased or reduced leading to dry mouth), gastrointestinal issues (nausea/vomiting, diarrhea, constipation), urinary problems, orthostatic dizziness, tachycardia, and increased sweating tendency. The other side effects category included weight changes, menstrual irregularities, galactorrhea, gynecomastia, alterations in sexual desire and function, and vaginal dryness.

Out of 152 patients, 97 (61.78%) experienced side effects, while 55 (36.18%) did not have any specified in their records. Among the 97 patients with side effects, 56 (57.73%) were women.

**Figure 3:**
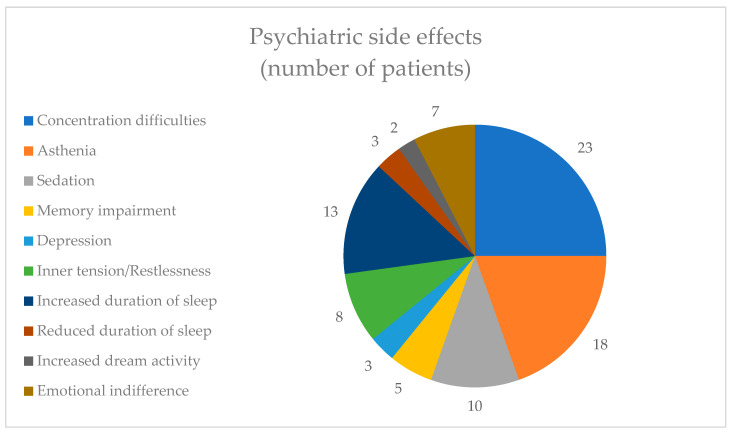
Psychiatric side effects.

Within the category of psychic side effects, the most commonly reported side effect was concentration difficulties, reported by 23 (23.71%) patients, followed by asthenia, reported by 18 (18.55%) patients. The least reported adverse effect from this category was increased dream activity, mentioned by only two (2.06%) patients (Figure 3).

**Figure 4:**
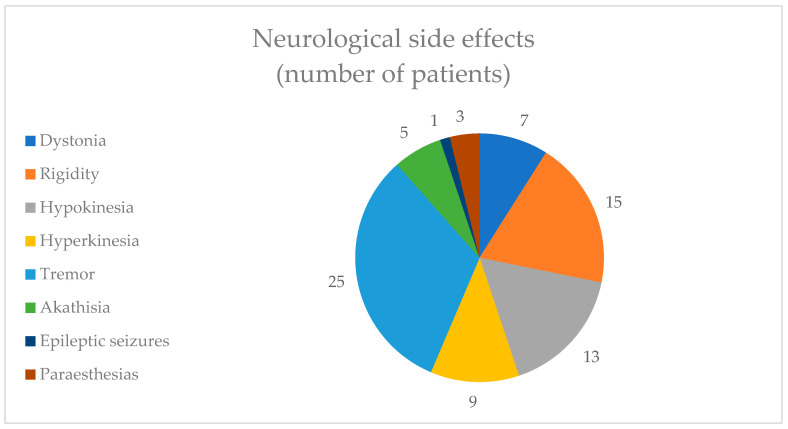
Neurological side effects.

In terms of neurological adverse effects, 25 (25.77%) patients reported tremors and 15 (15.46%) patients reported rigidity. Only one (1.03%) patient reported epileptic seizures (Figure 4).

**Figure 5:**
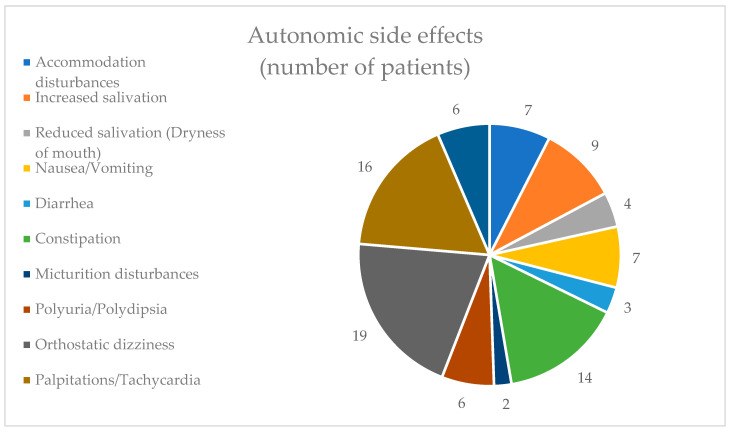
Autonomic side effects.

Among the autonomic side effects category, orthostatic dizziness was the most commonly experienced adverse effect, reported by 19 individuals (19.59%). This was followed closely by palpitations/tachycardia, noted by 16 individuals (16.49%). Micturition disturbances were the least encountered adverse effect, affecting only two individuals (2.06%) (Figure 5).

**Figure 6:**
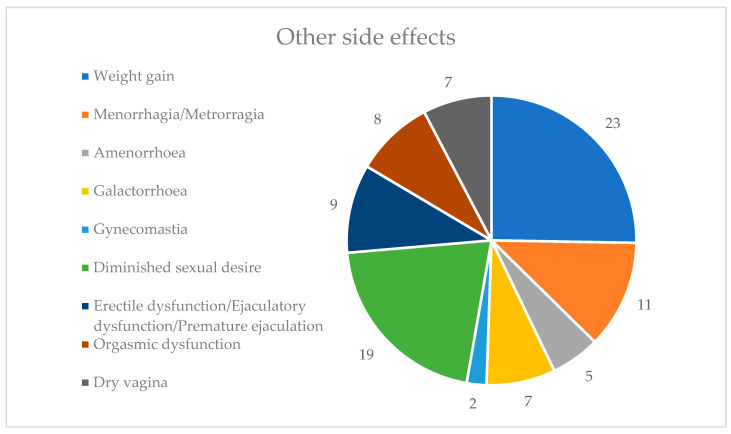
Other side effects.

In the other side effects category, weight gain was the most prevalent side effect, reported by 23 individuals (23.71%), followed by diminished sexual desire, experienced by 19 individuals (19.59%). Gynecomastia was the least reported side effect, affecting only two individuals (2.06%). None of the patients experienced weight loss or increased sexual desire (Figure 6). Given that a significant portion of patients experienced adverse effects in this category, many of which are symptoms associated with hyperprolactinemia, we hypothesized that elevated prolactin levels may induce these effects.

### 3.3. Prolactin Levels

We examined prolactin levels in all patients enrolled in the study. Out of the 152 patients, 82 (53.95%) had abnormal prolactin levels. Among those 82 patients with abnormal levels, 30 (36.58%) were men and 52 (63.41%) were women. 

Table 4 presents the characteristics of patients with elevated prolactin levels. Among men with elevated prolactin levels, risperidone is the most commonly prescribed antipsychotic, at 12 cases (40%), while among women with elevated prolactin levels, olanzapine is the most common, at 32 cases (61.54%). None of the patients receiving treatment with aripiprazole exhibited high levels of prolactin.

**Figure 7:**
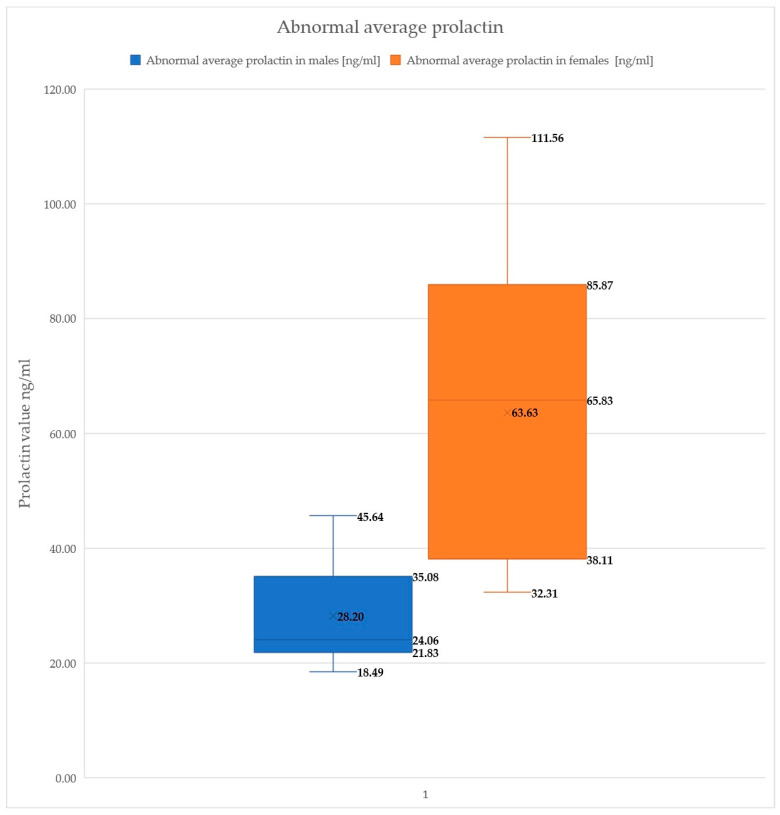
Abnormal prolactin levels.

In male patients with abnormal prolactin levels, the lowest recorded value was 18.49 ng/mL and the highest was 45.64 ng/mL. For female patients with abnormal prolactin levels, the lowest value observed was 32.31 ng/mL and the highest was 111.56 ng/mL (Figure 7).

### 3.4. CT Scan Results

In all 152 cases, the CT examinations revealed no presence of prolactinomas or other brain tumors. However, the results align with a prior study conducted by our team, indicating notable findings concerning brain morphology [34]. Specifically, patients exhibited the widening of the frontal horns and lateral ventricles, along with early indications of atrophy. Additionally, 7% of the patients presented with sinus issues, including sinusitis and nasal polyps.

**Figure 8:**
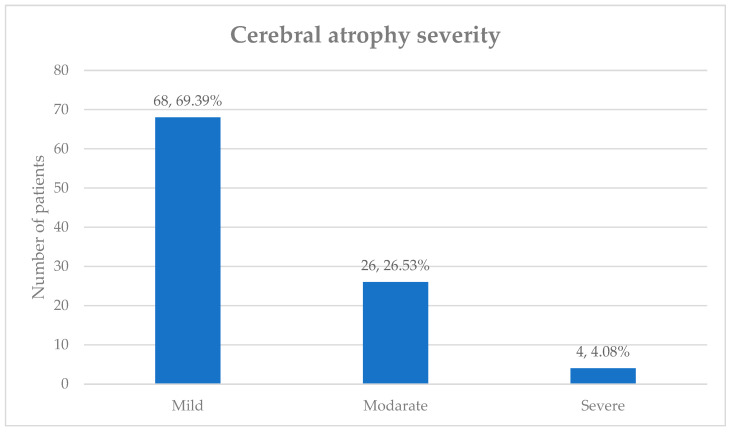
The severity of cerebral atrophy.

Atrophy was observed in a significant number of patients (*n* = 98, 64.47%), despite their young age. The frontal (*n* = 72, 73.47%) and temporal (*n* = 56, 57.14%) lobes were the most commonly affected regions, with many patients exhibiting atrophy in multiple areas. The severity of atrophy, presented in Figure 8, was categorized as mild, moderate, or severe. The majority of patients had mild atrophy (*n* = 68, 69.39%), followed by 26 (26.53%) with moderate atrophy, and only 4 (4.08%) with severe atrophy.

## 4. Discussions

This study aimed to explore the presence of brain tumors, prolactinomas, and other brain abnormalities in patients with schizophrenia receiving treatment with second-generation antipsychotics. Fortunately, neither brain tumors nor prolactinomas were detected. This finding aligns with previous research indicating that medication-induced hyperprolactinemia is more common than prolactinoma in individuals undergoing antipsychotic therapy. It supports the broader understanding that while antipsychotic medications can elevate prolactin levels, resulting in hyperprolactinemia, the development of actual prolactinomas is rare among this population [35].

Cortical atrophy was found in a significant number of patients in our study, which is consistent with findings reported in other studies [36]. The presence of cortical atrophy from the early stages of the disease may explain the suboptimal response in patients with schizophrenia, despite treatment with second-generation antipsychotics. This underscores cortical atrophy as a prominent feature in individuals with schizophrenia, highlighting its potential impact on disease progression and treatment outcomes.

A significant proportion of cases exhibiting hyperprolactinemia were detected in our study, including patients who displayed no symptoms associated with this condition. Notably, the highest incidence of hyperprolactinemia was observed among patients receiving treatment with risperidone and olanzapine. This finding is consistent with a study conducted by Turrone et al., which reported that the administration of risperidone and olanzapine leads to dose-dependent increases in prolactin levels [37].

In our patient cohort, a significant number experienced adverse effects that are indicative of symptoms associated with hyperprolactinemia. These findings align with previous research in the field [38]. Weight gain emerged as one of the most prevalent adverse effects, consistent with findings reported by Farhat et al. and Sengupta et al. [39,40]. The occurrence of adverse effects among psychiatric patients may vary based on factors such as age, gender, prescribed medications, and underlying health conditions. In our investigation, females exhibited a higher frequency of adverse effects, a trend observed in other studies as well [41,42]. This elevated risk among females could be explained by various factors, including gender-related disparities in drug metabolism, response, genetics, immune function, hormonal influences, and variations in medication use, such as contraceptives, compared to males [42].

The extended use of antipsychotic drugs known to elevate prolactin levels has been associated with various health concerns, including infertility, reduced bone mineral density, and an increased risk of fragility fractures in individuals with severe mental illness [43,44,45]. Moreover, in women, there is a heightened likelihood of breast cancer [46]. Gynecomastia and amenorrhea are also potential long-term side effects associated with prolonged antipsychotic use [47,48]. Consequently, the effective management of prolactin levels is important in these patients. The initial and most practical step involves repeating the analysis to prevent the possibility of any laboratory errors.

Another approach could involve reducing the dosage of antipsychotic medication, although this option carries certain disadvantages. Lowering the dosage of antipsychotic medication in individuals with schizophrenia may potentially exacerbate symptoms, necessitating hospitalization. Studies have linked relapses in schizophrenia to a gradual decline in brain volume [49]. The consequences of relapse in individuals with schizophrenia include an increased risk of self-harm and harm to others, heightened distress for both patients and their families, strains on friendships and relationships, interruptions in education and vocational pursuits, diminished patient autonomy, increased social stigma, elevated economic burdens associated with treatment, and greater risks of not returning to baseline functioning or displaying resistance to treatment [50,51].

Switching antipsychotic medications with one that has minimal impact on prolactin levels, such as aripiprazole, lurasidone, or ziprasidone, is another option [52]. However, switching antipsychotic medications in patients with schizophrenia can present several disadvantages. Firstly, it may lead to a period of instability or exacerbation of symptoms during the transition phase, potentially requiring additional monitoring or hospitalization. Secondly, there is the risk of encountering adverse effects specific to the new medication, which may vary from patient to patient and could necessitate further adjustments. Additionally, some patients may experience resistance or poor response to the new medication, resulting in inadequate symptom control. Moreover, changes in medication can disrupt the therapeutic alliance between patients and physicians, impacting treatment adherence and engagement in care [53]. 

Our research highlights the importance of integrating prolactin testing into the routine monitoring regimen for individuals diagnosed with schizophrenia. It emphasizes that effectively managing hyperprolactinemia is important in reducing its long-term risks. Consistent monitoring and ongoing consultations with healthcare professionals are essential for tracking progress and making necessary treatment adjustments, thereby minimizing the potential long-term consequences associated with this condition. Currently, prolactin evaluation is not routinely performed in all hospitals in Romania, but it should become mandatory.

This study has limitations due to its reliance on documentary sources, leading to a relatively small sample size of patients. This limitation arises because only patients with thorough and comprehensive records, containing all necessary information, were considered for inclusion. Additionally, the study’s specific focus on young individuals further reduces the sample size, potentially limiting the generalizability of the findings to larger populations. 

The correlation between structural changes and the type of antipsychotic used could not be established due to the absence of important data. Specifically, information regarding DUP (duration of untreated psychosis) and prior treatments (type, doses, duration, etc.) was not included in the analysis. Each of these variables is known to potentially influence structural brain changes, underscoring their importance in understanding the relationship between antipsychotic treatment and neuroanatomical alterations [54,55]. This is one of the limitations of this study.

Another limitation is the lack of information on patients who are on LAI treatments, are in clinical remission, and were not included in the evaluation. During the pandemic, hospitalizations for administering LAI treatments were suspended due to restrictions.

Despite facing initial challenges due to COVID-19 pandemic-related restrictions, the imaging evaluations requested during that period provided a comprehensive assessment of the patients, contributing strength to this study.

## 5. Conclusions

Despite the challenges caused by unhealthy lifestyles and limited access to medical investigations due to the specificity of schizophrenia, the incidence of brain tumors and prolactinomas in individuals with this condition appears to be relatively low. However, the treatment regimen for schizophrenia, particularly the use of antipsychotic medications, requires careful monitoring. The regular assessment of prolactin levels is important to promptly identify and prevent any adverse effects these medications may have. Elevated prolactin levels can lead to significant health issues, including reproductive, metabolic, and bone density problems. Thus, incorporating prolactinemia monitoring into routine clinical practice can help manage these potential side effects more effectively.

Although there are costs associated with the screening procedures, these should become standard practice in the care of individuals with schizophrenia. Regular screenings can play a role in the early detection and management of comorbid conditions, thereby reducing morbidity and mortality rates among this population. This approach is important for improving both the life expectancy and overall quality of life for individuals with schizophrenia.

## Figures and Tables

**Table 1:** Demographics and clinical characteristics of the study population.

Parameters			
Number of patients	152		
Male gender (*n*;%)	57; 37.5%		
Mean age (years ± SD)	42.79 ± 11.66		
Illness duration (years ± SD)	17.89 ± 11.56		
Number of episodes	25.19 ± 13.56		
Smoker (*n*; %)	93; 61.18%		
Antipsychotic medication			
	Number, %	Mean dose (mg) ± SD	Chlorpromazine equivalent (mg)
Amisulpride (*n*; %)	4; 2.63%	450 ± 100	450
Aripiprazole (*n*; %)	9; 5.92%	16.12 ± 7.41	214.94
Clozapine (*n*; %)	4; 2.63%	400 ± 70.71	400
Olanzapine (*n*; %)	70; 46.05%	14.5 ± 4.43	290
Quetiapine (*n*; %)	6; 3.94%	566.66 ± 150.55	755.55
Paliperidone (*n*; %)	3; 1.97%	8 ± 1.73	400
Risperidone (*n*; %)	56; 36.84%	3.33 ± 1.61	333

**Table 2:** Antipsychotic treatment by gender.

Antipsychotic Medication	Male *n* = 57	Female *n* = 95	*p* Value
Amisulpride (*n*; %)	2, 3.51%	2, 2.11%	0.60
Aripiprazole (*n*; %)	0	9, 9.47%	-
Clozapine (*n*; %)	2, 3.51%	2, 2.11%	0.60
Olanzapine (*n*; %)	25, 43.86%	45, 47.37%	0.67
Quetiapine (*n*; %)	3, 5.26%	3, 3.16%	0.52
Paliperidone (*n*; %)	2, 3.51%	1, 1.05%	0.29
Risperidone (*n*; %)	23, 40.45%	33, 34.73%	0.48

**Table 3:** Antipsychotic chlorpromazine equivalents.

Antipsychotic	Dose Equivalent
Chlorpromazine	100 mg
Amisulpride	100 mg
Aripiprazole	7.5 mg
Clozapine	100 mg
Olanzapine	5 mg
Quetiapine	75 mg
Paliperidone	2 mg
Risperidone	1 mg

**Table 4:** Characteristics of patients with elevated prolactin levels.

	Male		Female	*p* Value
Number of patients	30		52	-
Mean age (± SD)	37.67 ± 7.98		39.85 ± 6.52	0.25
Antipsychotic treatment				
	Male		Female	
	Number of patients (*n*; %)	Mean dose (mg) ± SD	Number of patients (*n*; %)	Mean dose (mg) ± SD
Amisulpride	1; 3.33%	-	2; 3.85%	500 ± 141.42
Aripiprazole	0	-	0	-
Clozapine	2; 6.67%	416.67 ± 160.73	0	-
Olanzapine	11; 36.67%	16.82 ± 4.04	32; 61.54%	15.74 ± 4.98
Quetiapine	3; 10%	500 ± 141.42	2; 3.85%	500 ± 141.42
Paliperidone	1; 3.33%	-	1; 1.92%	-
Risperidone	12; 40%	4.67 ± 1.3	15; 28.84%	4.8 ± 1.01

## Data Availability

The datasets are not publicly available due to hospital policies. The datasets used and/or analyzed during the current study are available from the corresponding author upon reasonable request. The approval document from the ethics committee for conducting the study is available upon request.
